# E-cigarette-Induced Pulmonary Inflammation and Dysregulated Repair are Mediated by nAChR α7 Receptor: Role of nAChR α7 in ACE2 Covid-19 receptor regulation

**DOI:** 10.21203/rs.2.23829/v2

**Published:** 2020-05-18

**Authors:** Qixin Wang, Isaac Sundar, Dongmei Li, Joseph Lucas, Thivanka Muthumalage, Samantha McDonough, Irfan Rahman

**Affiliations:** University of Rochester; University of Rochester Medical Center

**Keywords:** E-cig exposure, nAChR α7, Inflammation, Dysregulated repair

## Abstract

Electronic cigarette (e-cig) vaping is increasing rapidly in the United States, as e-cigs are considered less harmful than combustible cigarettes. However, limited research has been conducted to understand the possible mechanism that mediate, toxicity and pulmonary health effects of e-cigs. We hypothesized that sub-chronic e-cig exposure induces inflammatory response and dysregulated repair/extracellular matrix (ECM) remodeling, which occur through the α7 nicotinic acetylcholine receptor (nAChR α7). Adult wild-type (WT), nAChRα7 knockout (KO), and lung epithelial cell-specific KO (nAChRα7 CreCC10) mice were exposed to e-cig aerosol containing propylene glycol (PG) with or without nicotine. Bronchoalveolar lavage fluids (BALF) and lungs tissues were collected to determine e-cig induced inflammatory response and ECM remodeling, respectively. Sub-chronic e-cig exposure with nicotine increased the inflammatory cellular influx of macrophages and T-lymphocytes including increased pro-inflammatory cytokines in BALF and increased ACE2 Covid-19 receptor, whereas nAChR α7 KO mice show reduced inflammatory responses associated with decreased ACE2 receptor. Interestingly, matrix metalloproteinases (MMPs), such as MMP2, MMP8, and MMP9 were altered both at the protein and mRNA transcript levels in female and male, but WT mice exposed to PG alone showed a sex-dependent phenotype. Moreover, MMP12 was increased significantly in male mice exposed to PG with or without nicotine in a nAChR α7-dependent manner. Additionally, sub-chronic e-cig exposure with or without nicotine altered the abundance of ECM proteins, such as collagen and fibronectin significantly in a sex-dependent manner, but without the direct role of nAChR α7 gene. Overall, sub-chronic e-cig exposure with or without nicotine affected lung inflammation and repair responses/ECM remodeling, which were mediated by nAChR α7 in a sex-dependent manner.

## Introduction

E-cigarettes (e-cigs) are often considered as a safer alternative to combustible cigarettes, as well as a method for quitting traditional cigarette smoking [1, 2]. The e-cig delivery system is based on tanks or cartridges that are loaded with e-cig liquid (e-liquid), which is then aerosolized, with inhalation delivering the aerosol to the lungs. Usually, e-liquids are composed of propylene glycol (PG) and/or vegetable glycerin (VG), with varying concentrations of nicotine (up to 100 mg/mL). Flavoring chemicals are added as additives in e-liquids and pods that enhance the taste and reduce throat hit, however they tend to attract younger generation unexpectedly [3, 4]. While PG and VG are considered safe in food-grade products, adverse health effects have been reported from these substances when they serve as e-cig vehicles [5]. Previous studies have shown that the amount of nicotine delivered by e-cigs is much higher than the amount delivered via cigarette smoke [6, 7]. We have shown that inflammation and extracellular matrix (ECM) remodeling/dysregulated repair are altered by acute exposure to e-cigs, with or without nicotine [8]. Considering that e-cig vaping is often a long-term habit, research on the chronic effects of e-cig aerosol exposure is essential to understand the mechanism resulting in augmented inflammatory responses and ECM remodeling, which are fundamental changes that occurs during early stages of most chronic lung diseases, such as idiopathic pulmonary fibrosis (IPF) and chronic obstructive pulmonary disease (COPD) [9, 10].

In our previous studies, acute exposure to e-cig aerosol containing flavoring chemicals was shown to cause lung inflammation, oxidative stress, and dysregulated repair [8, 11]. Further, nicotine exposure could cause suppression of immune response resulting in augmented lung inflammation and injury following viral infection [12]. Nicotinic Acetylcholine Receptors (nAChRs) are largely responsible for activation of acetylcholine neurotransmitter signaling pathways in the central nervous system (CNS) [13, 14]It has been postulated that angiotensin-converting enzyme (ACE2), a Covid-19 receptor is regulated by nAChRs. The nAChRs are widely distributed in the CNS, so they can easily be activated by nicotine, initiating and reinforcing a rewarding feedback loop which might induce nicotine addiction [15, 16]. Interestingly, lung nAChRs activation could help inhibit the inflammation caused by lipopolysaccharides (LPSs) and the receptor knockouts (KOs) that promote inflammatory processes [17, 18]. However, as one of the nAChR-agonists, nicotine could initiate receptor-related pathways, and nicotine aerosol inhalation also induce inflammation [11], and regulate ACE2 receptor via nAChR. Other chemicals may induce activation of nAChRs, and they may also trigger feedback-loops, similar to flavoring chemicals [19–21]. However, there are limited studies that directly describe about the role of dysregulated repair induced by e-cig vapors with or without nicotine in a nAChRα7-dependent manner.

A common vehicle used for nicotine delivery in e-cigs is PG and/or VG. PG (C_3_H_8_O_2_) is a colorless and odorless organic solvent that is an FDA-approved, food-grade chemical generally recognized as safe (GRAS). This is one of the reasons why people originally thought to use PG as a nicotine carrier in e-liquids. Previous study has shown that PG aerosols produce different byproducts, some of which might lead to cancer [22]. Many e-cigs with or without nicotine are commercially available, but the understanding of respiratory health risks of e-cig inhalation are poorly investigated.

In this study, we hypothesized that e-cig aerosol induce lung inflammation and dysregulated repair/ECM remodeling in a nAChR α7-dependent manner. Our results suggest that lung inflammatory responses and dysregulated repair/ECM remodeling induced by e-cig aerosol containing nicotine could potentially be related to the nAChR α7 signaling pathway. On the other hand, PG-induced lung dysregulated repair and inflammatory response occurs in a nAChR α7-independent manner.

## Methods

### Animals

Adult C57BL/6J (WT) mice and α7 nicotinic acetylcholine receptor knockout (nAChR α7 KO) mice were both purchased from Jackson Laboratory, weighing 25–35 grams and aged 3–4 months old. The nAChR α7 CreCC10 mice (clara/club-cell-specific nAChR α7 deletion) were generated by crossing nAChR α7 floxed mice (nAChR α7 floxed mutant) from Jackson Laboratory (donated by Dr. Jerry Yakel, NIEHS/NIH) with mice have the Cre recombinase transgene controlled by the CC10 promoter (C57BL/6J; from TJ Mariani, University of Rochester, Rochester, NY). Prior to e-cig exposure, mice were housed in the inhalation core facility at the University of Rochester for one week. All experiments performed in this study were in compliance with the standards set by the United States Animal Welfare Act. The Animal Research Committee (UCAR) approved the animal protocol at the University of Rochester Medical Center, Rochester, NY.

### Blood gas and exercise ability measurement

Blood gas, including pH, pressure of CO_2_ and O_2_, concentration of HCO_3_, TCO_2_, glucose (Glu) and hemoglobin (Hb), percentage of O_2_ and hematocrit (Hct) were analyzed by i-STAT system (i-STAT CG8+ cartridge, Cat# 03P88–25; Vet scan i-STAT 1 analyzer; Abaxis Global Diagnostics), as described previously [23]. Exercise capacity measurement was done using a motorized animal treadmill (Columbus Instruments) as described previously [24]. Running distance (meters) and running time (minutes) were recorded to present exercise tolerance. Mice stayed on the treadmill for 5 min before start of measurement. The treadmill began at 8.5 m/min with 0° incline for 9 min. Then, the speed was increased to 10 m/min with 5° incline for 3 min. After that, the speed was increased to 2.5 m/min every 3 min, and incline was increased to 5° every 9 min until the mouse gets exhausted, judged by observation of failure of running and continuous contact with the electric grid. Following this exercise, blood was collected by submandibular venipuncture for blood gas and cotinine measurement.

### E-cig device and e-liquid

The e-cig device used in this study was purchased from Joytech VTC mini (SciReq, Montreal, Canada). The atomizer/coil (0.15Ω) used for aerosolization of e-liquid was purchased from Kanger technology (Shenzhen, China). All the other components of the e-cig exposure chamber were purchased from SCIREQ, and all the components were cleaned after exposure every day. The atomizer/Coil was replaced on a weekly basis to avoid overheating and generation of carbon monoxide. The e-liquids used in this study that contains PG alone and PG with nicotine (25 mg/mL), were purchased from xtremevaping.com.

### E-cig exposure

The e-cig exposure performed here has been described in our previous studies [25]. Briefly, the *in vivo* e-cig exposure was setup inside a fume hood and based on the SCIREQ InExpose e-cig extension smoking system. The e-cig exposure puffing profile used was based on realistic topographical data from e-cig users, with 3.3 sec/puff, 2 puffs/min and a 70mL puff volume [26]. The aerosolization of e-liquid was performed by using a 3^rd^ generation e-cig device (Joytech eVIC VTCmini), which was controlled by the SCIREQ flexiware software (V8.0). The whole-body exposure was done for a total of 2 hrs/day, 5 days/week, for 30 days. During e-cig exposures, temperature, humidity, oxygen and carbon dioxide percentages were monitored along with carbon monoxide levels in the chamber. The e-cig aerosol generated was passed through the condensing chamber and pumped into the mixing chamber with a flowrate of 1.0 L/min. The vapor was diluted with air in the mixing chamber and then delivered into the whole-body exposure chamber where mice were separated by dividers. Simultaneously, the e-cig aerosol in the exposure chamber was exhausted by another pump with a flowrate of 2.0 L/min. Both the pumps were calibrated and adjusted each time before exposure. Pumps were cleaned at the end of each exposure to minimize the effects of nicotine residues. Mice were divided into air (control), PG, and PG with nicotine groups, each with an equal number of males and females, for both the WT and nAChR α7 KO conditions. Air group mice stayed in the inhalation facility in a similar environment during the 30 day exposure. Serum cotinine levels measured by ELISA (Calbiotech) were ~500 ng/mL for the PG with nicotine group, and ~20 ng/mL for the PG only group, due to nicotine residue in the pumps and exposure chambers which was difficult to fully clean (Additional File 1: Figure. S1). As expected, the air group showed no cotinine.

### Bronchoalveolar lavage fluid (BALF)

Mice were euthanized with Ketamine/Xylazine 24 hrs after the final exposure. Their tracheas were cannulated and their lungs were lavaged three times with 0.6 mL saline with 1% FBS (1.8 mL total). The recovered fluids were collected and spun down at 1000 × g for 10 min at 4°C for harvesting of BALF cells. The supernatant was stored at −80°C for future analysis. The BALF cells were re-suspended in 1.0 mL saline with 1% FBS and stained with acridine orange propidium iodide (AO/PI). Total cell counts per mL were measured from AO/PI stained cells via cellometer.

### Inflammatory cell count

The resuspended BALF cells were used for immune-inflammatory cell counts with cell-type-specific labelled monoclonal antibodies. Total 1.0 × 10^5^ BALF cells were used for antibody labeling. Before antibody staining, all cells were blocked with purified anti-mouse CD16/32 (Cat# 50-163-432, Fisher Scientific) to prevent non-specific binding, and washed with PBS once. The cells were stained with F4/80 PE-conjugated antibody for macrophages (Cat# 123109, BioLegend), LY6B.2 Alexa fluor488-conjugated antibody for neutrophils (Cat# NBP213077AF488, Novus Biologicals), PE-Cyanine7 antibody for CD4a^+^ T-lymphocytes (Cat# 25-0041-82, Fisher Scientific), and APC conjugated Monoclonal Antibody for CD8a^+^ T-Lymphocytes (Cat# 17-0081-82, Fisher Scientific). The absolute cell numbers of macrophages, neutrophils, and CD4a^+^/CD8a^+^ T-lymphocytes were determined by multiplying the percentage of cells by the total cell counts. Flow cytometry was performed using the Guava® easyCyte™ flow cytometer (Millipore Sigma) and analyzed using Guava® InCyte™ software.

### Measurement of pro-inflammatory cytokines by Luminex in BALF

To measure the pro-inflammatory cytokines present in BALF, a Bio-Plex Pro mouse cytokine 23-plex immunoassay kit (Cat#: M60009RDPD, BioRad) was used, according to the manufacturer’s instructions. Briefly, the diluted magnetic beads were placed into the assay plate and rinsed with wash buffer. The BALF samples and standards were then added into the wells, and shaken at 850 rpm at room temperature for 30 min. After sample incubation, the plates were washed with wash buffer 3 times, then the detection antibody was added and the plates incubated for 30 min at room temperature, shaking at 850 rpm. Next, plates were washed with wash buffer 3 times, and SA-PE was added for 10 min at room temperature, shaking at 850 rpm. After this step, the plates were washed 3 times with wash buffer, and the beads were resuspended in assay buffer for reading. Results are determined via the Luminex flexmap 3d (Luminex Corp.)

### Protein isolation

Lung tissues harvested from the animals during sacrifice were stored at −80°C for future analysis. Frozen lungs were homogenized mechanically in RIPA buffer with protease inhibitor (Cat#: 78440, ThermoFisher Scientific). After homogenization, lysates were kept on ice for 45 min, followed by centrifugation at 15,000 × g for 30 min at 4°C. The supernatant was collected and protein concentration was quantified using the Pierce BCA Protein Assay Kit (Cat#: 23227, ThermoFisher Scientific), based on the manufacturer’s protocol.

### Western blot

The protein samples from lung homogenates were running through SDS-polyacrylamide electrophoresis gels (SDS-PAGE: 10%) with 20 μg of protein in each lane. After electrophoresis, the gel was transferred onto a nitrocellulose membrane (Cat# 1620112, BioRad). The membrane was washed with tris-buffered saline containing 0.1% Tween 20 (TBS-T) for 15 min, then blocked with 5% non-fat dry milk for 1 hr at room temperature. Then, the membranes were probed with primary antibodies 16 hr at 4°C: anti-MMP2 (1:1000, ab92536, Abcam); anti-MMP9 (1:1000, ab38898, Abcam); anti-MMP8 (1:1000, ab81286, Abcam); anti-MMP12 (1:1000, NBP2–67344, Novus Biologicals); anti-Collagen 1α1 (1:1000, NBP1–30054, Novus Biologicals); anti-Collagen 1α2 (1:500, NBP1–57987, Novus Biologicals); anti-fibronectin (1:2000, ab45688, Abcam); and anti-PAI-1 (1:1000, ab182973, Abcam),anti-ACE2 (1:1000, ab15348, Abcam), and the appropriate secondary antibody (Goat-Anti-Rabbit, 1:10000, Cat# 1706515, BioRad) for 1 hr at room temperature. After each antibody incubation, membrane was washed 4 times in TBS-T at room temperature, 15 min per wash. The luminescence signals were developed using chemiluminescence substrate (Perkin Elmer, Waltham, MA). The membrane exposure and band intensities were detected via the Bio-Rad ChemiDoc MP imaging system (Bio-Rad Laboratories, Hercules, CA, USA). Protein quantification was done by densitometry analysis, and β-actin (1:2500, ab20272, Abcam) was used as housekeeping control.

### RNA isolation and Nanostring quantification

Frozen lung tissues from −80°C were used for RNA isolation. Lung tissue (~100 mg) per sample was homogenized in Trizol for 20s. The RNA was isolated using the Direct-zol™ RNA Miniprep Plus assay kit (Cat# R2073, Zymo Research). Isolated RNA was quantified and checked for quality by spectrophotometer (ND-1000, NanoDrop Technologies, Wilmington, DE, USA). RNA samples were aliquoted and sent out to Nanostring facility for analysis of genes using nCounter Mouse Myeloid Innate Immunity v2 Panel (NanoString Technologies, Inc. Cat# XT-CSO-MMII2–12).

### Statistical analysis

The statistical analysis was done by either one-way ANOVA or student’s t-test, followed by Tukey’s multiple comparisons in GraphPad Prism software (Version 8.0, La Jolla, CA). Results were presented as the mean ± SEM. *P* < 0.05 was considered statistically significant.

## Results

### Sub-chronic e-cig exposure altered exercise capacity and blood-gas saturation

After 30 days e-cig exposure, WT (floxed) mice showed no difference in exercise capacity among the groups (Additional File 1: Figure S2). However, nAChR α7 deficient (KO) mice showed less capacity to run when exposed to PG with nicotine compared to air control and PG alone groups. Similar results were observed in nAChR α7 lung epithelial cell-specific conditional KO mice exposed to PG with nicotine show reduced exercise capacity compared to air control and PG alone groups. E-cig aerosol with nicotine exposure in nAChR α7 KO show lowered exercise capacity indicating the possible role of nAChR α7 in the lung epithelium. Blood pH, O_2_ pressure, concentrations of HCO_3_ and CO_2_ were shown to be dysregulated when WT mice were exposed to PG with nicotine compared to PG alone, while no difference was observed between WT and nAChR α7 KO mice exposed to PG with or without nicotine (Additional File 2: Table S1).

### Sub-chronic e-cig exposure induces inflammatory cellular influx in the mouse lung

**Figure 1:**
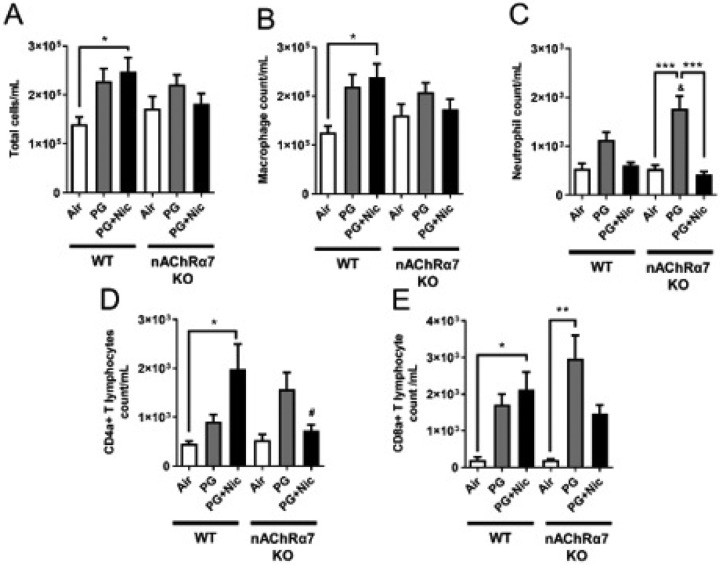
Sub-chronic e-cig exposure augments inflammatory cell influx in BALF. Differential inflammatory cell counts were measured in BALF from mice exposed to air, PG or PG with nicotine (PG+Nic) for 30 days (2 hrs/day). (A) Total inflammatory cell counts were analyzed by Cellometer using AO/PI staining. Differential inflammatory cell counts were determined as percentages by flow cytometry. Absolute cell counts for (B) F4/80+ macrophages, (C) Ly6B.2+ neutrophils, (D) CD4a+ T-lymphocytes, and (E) CD8a+ T-lymphocytes were normalized to the total cell counts. Data are shown as mean ± SEM (n=4–6/group; equal number of male and female mice). * P < 0.05, ** P < 0.01 between groups; & P < 0.05, compared to PG exposed WT group; # P < 0.05, compared to PG+Nic exposed WT group.

The total cell counts were increased in WT mice exposed to PG with or without nicotine relative to respective air group, and total cell counts in WT mice exposed to PG with nicotine increased significantly compared to air and PG alone groups (Figure 1A). The nAChR α7 KO mice showed no change in total cell counts when exposed to PG with nicotine (Figure 1A). However, the total cell counts were increased but not significant compared to respective control group (Figure 1A). Macrophage counts followed a similar trend as observed in total cell counts (Figure 1B). WT and nAChR α7 KO mice exposed to PG with nicotine showed no change in neutrophil counts in BALF (Figure 1C). Additionally, CD4a^+^ and CD8a^+^ T-lymphocyte counts were significantly increased when WT mice were exposed to PG with nicotine, and loss of nAChR α7 prevents increase in T-lymphocytes (Figure 1D–E). PG-exposed nAChR α7 KO mice showed higher levels of neutrophils and CD4a^+^/CD8a^+^ T-lymphocytes than PG-exposed WT mice (Figure 1C–E).

### Sub-chronic e-cig exposure augments influx of pro-inflammatory cytokines in BALF

**Figure 2:**
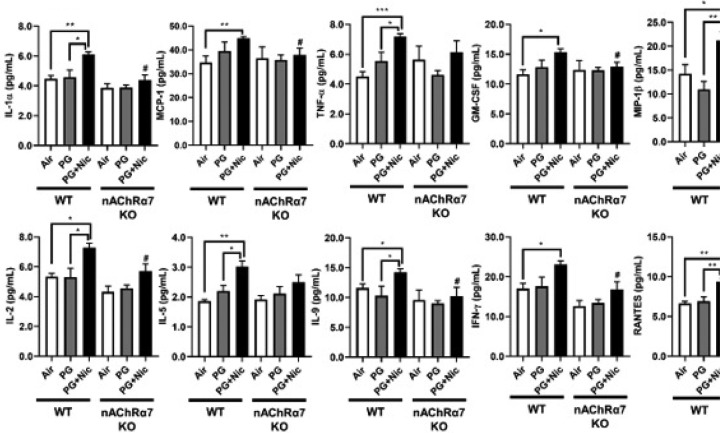
Sub-chronic e-cig exposure-induced pro-inflammatory mediators in BALF. Bio-Plex Pro mouse cytokine 23-plex assay kit (Bio-Rad) was used to determined levels of pro-inflammatory cytokines/chemokines in BALF from mice exposed to e-cig with or without nicotine for 30 days (2 hrs/day). Significant changes were found in cytokines related to macrophages (IL-1α, MCP-1, TNF-α, GM-CSF, and MIP-1β) and T-lymphocytes (IL-2, IL-5, IL-9, RANTES, and IFN-γ). Data are shown as mean ± SEM (n=6–10/group; equal number of male and female mice). * P < 0.05, ** P < 0.01, *** P < 0.001, compared to air control; # P < 0.05, compared to PG+Nic exposed WT mice.

We next determined the level of pro-inflammatory cytokines in BALF after sub-chronic e-cig exposure. Most of the pro-inflammatory cytokines in BALF were significantly increased in WT mice exposed to PG with nicotine compared to PG alone and air control (Figure 2 and Additional File 2: Table S2). We have noticed that macrophage mediated pro-inflammatory cytokines: IL-1α, MCP-1, and GM-CSF were significantly increased in WT mice exposed to PG with nicotine, and nAChR α7 KO blocks the release of those mediators (Figure 2). Similarly, lymphocytes-related cytokines: IL-2, IL-9, IFNγ, and RANTES showed nAChR α7-dependent reduction in cytokine levels (Figure 2). Additionally, TNFα, MIP-1β, IL-1β and IL-5 were increased in PG with nicotine in WT mice, but not in nAChR α7 KO mice exposed to PG with nicotine. The remaining cytokines IL-3, IL-4, IL-10, IL-12p40, IL-17A, G-CSF, and MIP-1α were not affected among any of the exposure groups in WT and nAChR α7 KO mice compared to respective air control (Additional File 2: Table S2).

### Sub-chronic e-cig exposure alters mRNA transcript levels of inflammation and dysregulated repair response genes in WT and nAChR α7 KO mice

To further determine whether sub-chronic e-cig exposure mediated inflammatory responses and dysregulated repair/ECM remodeling was caused by altered expression of myeloid innate immune response target genes (~734 genes), we performed gene expression analysis using nCounter Mouse Myeloid Innate Immunity Panel (Nanostring Technologies, Inc.). The pairwise comparison between PG alone exposed WT and nAChR α7 KO mice, revealed 21 genes were differentially expressed compared to respective air control (Figure 7B). Additionally, pairwise comparison analysis among PG with nicotine exposed WT and nAChR α7 KO mice revealed 89 genes differentially expressed when compared to respective air control (Figure 7B). Furthermore, pairwise comparison between PG with nicotine and PG alone groups in WT and nAChR α7 KO mice revealed 57 genes differentially expressed among them (Figure 7B).

**Figure 3:**
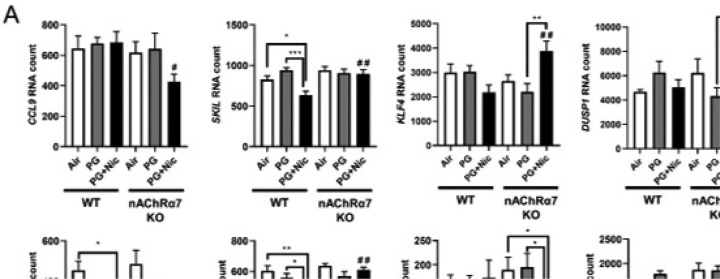

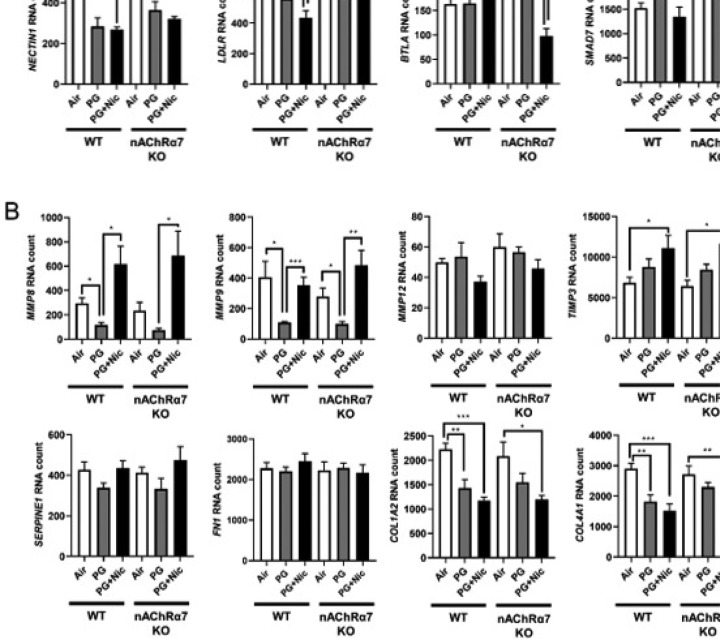
Sub-chronic e-cig exposure affects mRNA expression of myeloid and innate immune response target genes analyzed by Nanostring analysis. Mice were exposed to e-cig aerosol with or without nicotine for 30 days (2 hrs/day), and sacrificed 24 hrs after final exposure. RNA was isolated from lungs and screened via nCounter Mouse Myeloid Innate Immunity Panel using Nanostring analysis. Targets were selected based on significant differences, especially (A) PG+Nic exposed WT and nAChR α7 KO mice, or (B) ECM remodeling focused. RNA counts were normalized to multiple housekeeping genes and quantified using nSolver. Data are shown as mean ± SEM (n=6/group; equal number of male and female mice). * P < 0.05, ** P < 0.01, *** P < 0.001 between groups; # P < 0.05, # # P < 0.01 compared with PG+Nic exposed WT group.

WT mice showed significantly higher RNA counts indicative of increased mRNA transcript levels for multiple targets in PG with or without nicotine exposed groups, compared to air control (Additional File 1: Figure S3). When we compared WT mice exposed to PG alone, air control or PG with nicotine showed altered gene expression of inflammatory and ECM remodeling targets such as matrix metalloproteases (MMP9, and MMP8), S100A8, and collagens (COL17A1 and COL14A1) (Additional File 1: Figure S3A). When WT mice exposed to PG with nicotine were compared with air control, we found significant difference in transcript levels of inflammatory targets such as ARG1 and LPL (Additional File 1: Figure S3A). PG with nicotine exposure caused significant alterations in the expression of several target genes such as SMAD7, KLF4, CDH1, COL4A2, ICAM1, LDLR, IL1B, TLR5, NFKBIA, and CXCL2 among WT and nAChR α7 KO mice that belong to the broad categories inflammation and dysregulated repaired/ECM remodeling (Additional File 1: Figure S3B). Based on the above-mentioned gene expression analysis by Nanostring, we choose specific target genes for further analysis (Figure. 3).

Based on the Nanostring nCounter analysis, transcript levels of target genes that showed significant differences between WT and nAChR α7 KO mice following PG with nicotine exposure were selectively represented in Figure 3A. The gene expression levels of SKIL (Ski-like protein) and LDLR (Low-density lipoprotein receptor) were significantly decreased in WT mice exposed to PG with nicotine, but not nAChR α7 KO mice. However, the gene expression levels of CCL9 (Chemokine ligand 9), KLF4 (Kruppel-like factor 4), DUSP1 (Dual Specificity Phosphatase 1), BTLA (B and T Lymphocyte Associated), and SMAD7 (SMAD Family Member 7) were significantly increased only in nAChR α7 KO mice exposed to PG with nicotine compared to WT mice (Figure 3A). Finally, NECTIN1 gene expression in WT and nAChR α7 KO mice showed a decreased trend, but only WT mice exposed to PG with nicotine showed a significant reduction in the expression of NECTIN1 transcript compared to air control (Figure 3A).

### Sub-chronic e-cig exposure with nicotine in nAChR α7 deficient mice prevents dysregulation of p50/p105

**Figure 4:**
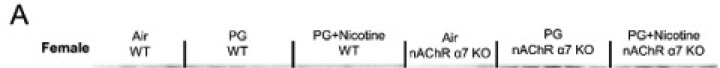

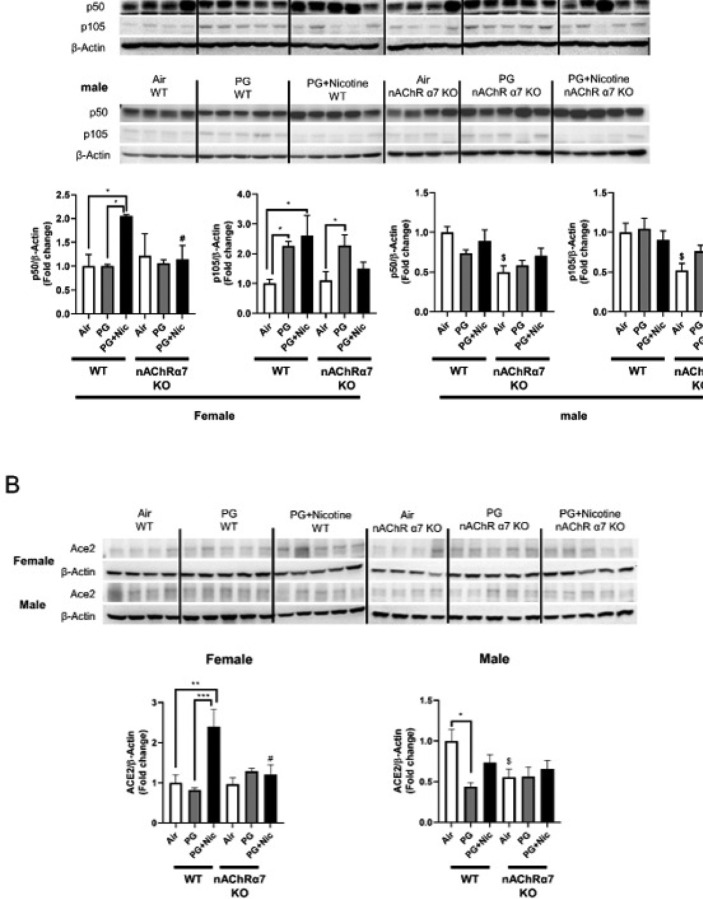
Sub-chronic e-cig exposure differentially affects the protein abundance of NF-κB subunits (p50/p105) and Angiotensin-converting enzyme (ACE2) in mouse lungs. The protein abundance of (A) p50/p105 and (B) Angiotensin-converting enzyme (ACE2) were measured in whole lung homogenates via Western blot. Representative blot images for female and male mice are shown. Densitometry analysis of individual blots was performed for p50/p105 and ACE2 for both female and male, and β-actin was used as an endogenous control. Data are shown as mean ± SEM. (n=4–5/group). * P < 0.05 significant compared between groups; $ P < 0.05, compared with air exposed WT group; # P < 0.05, compared with PG+Nic exposed WT group.

In order to determine the role of target molecules that contribute to e-cig exposure induced inflammatory response at the protein level, we measured the protein abundance of a NF-κB subunit (nuclear factor kappa-light-chain-enhancer; p50/p105). We have observed that the protein levels of both p50 and p105 were upregulated in WT female mice exposed to PG with nicotine, while nAChR α7 KO showed attenuation of p50 and p105 expression in the lungs (Figure 4A). However, there was no significant difference in male mice exposed to PG with or without nicotine compared to air control in both WT and nAChR α7 KO mice (Figure 4A). WT or nAChR α7 KO female mice exposed to PG alone showed similar upregulation of p105, indicating that PG alone could induce pro-inflammatory responses in a nAChR α7-independent manner (Figure 4A).

### Sub-chronic e-cig with nicotine exposure induced up-regulation of Angiotensin-converting enzyme (ACE2) in mouse lung

Recently, it has been shown that ACE2 (Covid-19 receptor) is a key point in migration of fibroblast to injured locations in lungs, and ACE2 is involved in lung ECM remodeling. We determined the protein abundance of ACE2 (Figure 4B). We have noticed that PG with nicotine has increased the protein abundance, whereas nAChR α7 deletion attenuated the dysregulation in female. While in male, there was no difference between PG with nicotine exposure and air control, PG exposure decreased the ACE2 protein level, and nAChR α7 knockdown lowered the baseline abundance of ACE2 in lungs.

### Sub-chronic e-cig exposure with or without nicotine induces dysregulated repair/ECM remodeling in a nAChR α7-independent manner

**Figure 5:**
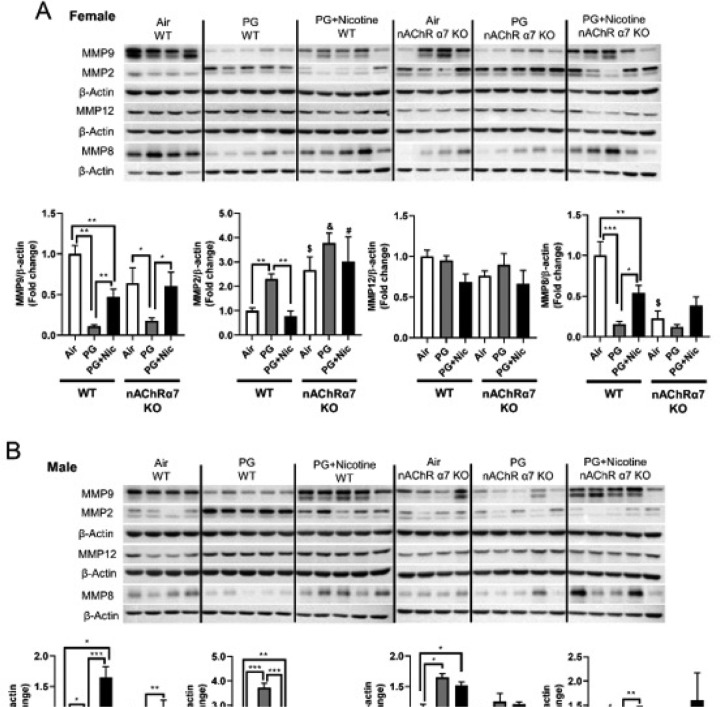

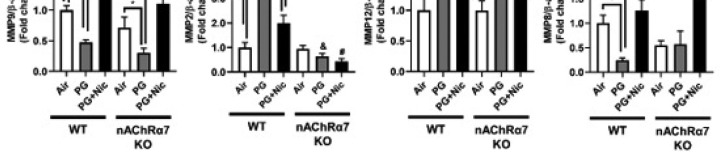
Sub-chronic e-cig exposure affects protein abundance of matrix metalloproteinases (MMPs) in mouse lungs. Protein levels of several MMPs (MMP9, MMP2, MMP12, and MMP8) were measured in whole mouse lung homogenates via Western blot. Representative blot images and densitometry analyses for female (A) and male (B) mice are shown. β-actin was used as an endogenous control. Data are shown as mean ± SEM. (n=4–5/group). * P < 0.05 between groups; $ P < 0.05, compared with air exposed WT group; & P < 0.05, compared with PG exposed WT group. # P < 0.05, compared with PG+Nic exposed WT group.

**Figure 6:**
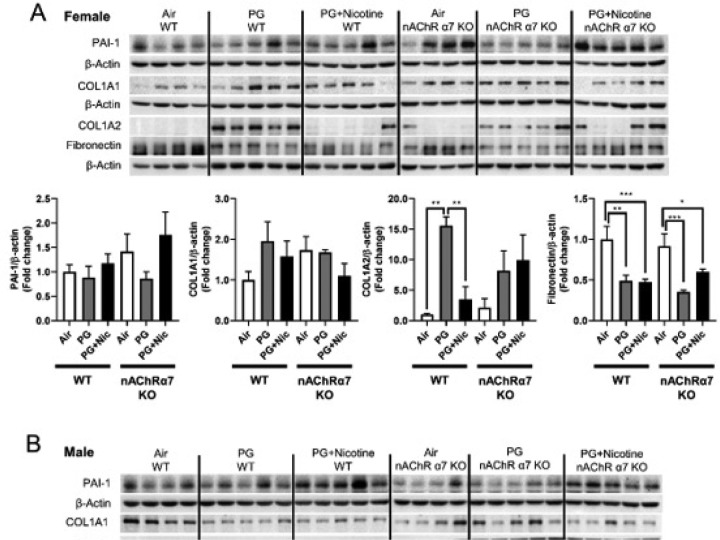

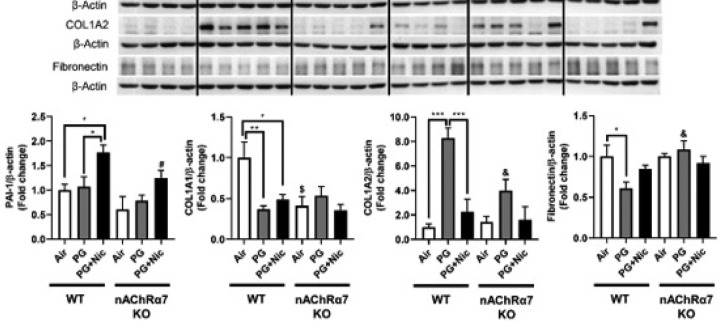
Sub-chronic e-cig exposure affects protein abundance of ECM-related markers in mouse lungs. The abundance of ECM proteins (PAI-1, COL1A1, COL1A2, and fibronectin) were measured in whole mouse lung homogenates via Western blot. Representative blot images and densitometry analyses for female (A) and male (B) mice are shown. β-actin was used as an endogenous control. Data are shown as mean ± SEM. (n=4–5/group). (* P < 0.05 between groups; $ P < 0.05, compared with air exposed WT group; & P < 0.05, compared with PG exposed WT group. # P < 0.05, compared with PG+Nic exposed WT group).

**Figure 7:**
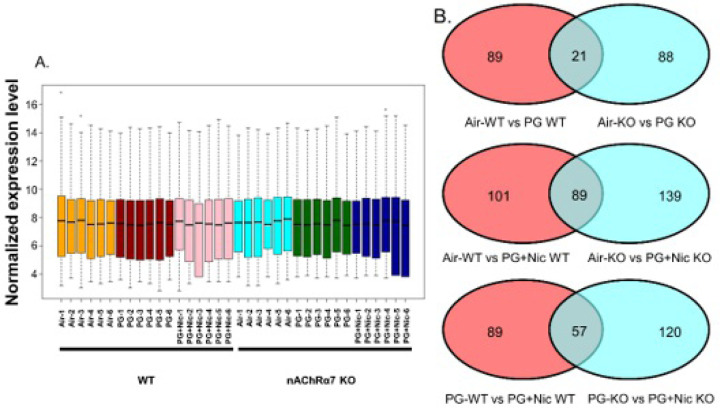
Sub-chronic e-cig exposure affects protein abundance of ECM-related markers in mouse lungs. The abundance of ECM proteins (PAI-1, COL1A1, COL1A2, and fibronectin) were measured in whole mouse lung homogenates via Western blot. Representative blot images and densitometry analyses for female (A) and male (B) mice are shown. β-actin was used as an endogenous control. Data are shown as mean ± SEM. (n=4–5/group). (* P < 0.05 between groups; $ P < 0.05, compared with air exposed WT group; & P < 0.05, compared with PG exposed WT group. # P < 0.05, compared with PG+Nic exposed WT group).

In our previous study, we have identified that acute e-cig exposure causes dysregulated repair/ECM remodeling in mouse lungs [8]. In this study, we were interested to investigate the role of dysregulated repair/ECM remodeling in sub-chronic e-cig exposed WT and nAChR α7 KO mice. We were specifically interested in selected MMPs and ECM remodeling markers to determine their effects following sub-chronic e-cig exposure both at the gene expression level (Figures 3B), and protein abundance (Figure 5–6).

PG exposed WT and nAChR α7 KO mice showed decreased expression of MMP8 and MMP9 compared to air control or PG with nicotine group (Figure 3B). WT and nAChR α7 KO mice when exposed to PG with nicotine showed significant increase in TIMP3, which is an inhibitor of MMPs (Figure 3B). ECM target genes fibronectin (FN1) or Plasminogen activator inhibitor-1 (PAI-1, gene code: SERPINE1) remain unaffected, while genes that modulate collagen expression (COL1A2 and COL4A1) was significant decreases in PG with or without nicotine exposed WT and nAChR α7 KO mice.

Based on the gene expression changes in dysregulated repair markers, we measured the protein abundances of the same in e-cig exposed WT and nAChR α7 KO mice. Results from immunoblot analysis correlated with gene expression of MMP9 and MMP8 in both female and male mice (Figure 5A–B). Decreased MMP8 and MMP9 were also observed in PG-exposed WT and nAChR α7 KO mice compared to PG with nicotine and air control (Figure 5A–B). MMP2 protein abundance was increased in WT male and female mice exposed to PG alone, compared to air control and PG with nicotine-exposed mice. Moreover, nAChR α7 KO female mice show increased MMP2 protein abundance at baseline, while no change was observed in male mice (Figure 5A–B). The protein levels of MMP9, MMP2, and MMP12 were increased in WT male mice exposed to PG with nicotine, while nAChR α7 KO inhibits protein abundance of MMPs (Figure 5B). MMP8 protein levels were comparable in female and male mice and nAChR α7 KO showed decreased protein abundance of MMP8 at baseline (Figure 5A–B).

ECM proteins/regulator such as collagens, fibronectin, and PAI-1, at the protein levels were measured in WT and nAChR α7 KO mouse lungs (Figure 6). The protein abundance of PAI-1 showed no difference among air, PG alone and PG with nicotine exposed WT and nAChR α7 KO female mice (Figure 6A). Whereas, PAI-1 was increased in WT male mice exposed to PG with nicotine, but was reduced in nAChR α7 KO male mice (Figure 6B). Furthermore, two different subunits of type 1 collagens (COL1A1 and COL1A2) were differentially expressed at the protein level among the experimental groups. WT and nAChR α7 KO female and male mice showed increased expression of COL1A2 when exposed to PG alone (Figure 6). However, COL1A1 protein abundance was reduced in WT male mice exposed to PG with or without nicotine, and nAChR α7 deficiency further decreased the baseline of COL1A1 levels in male mice. There is no change in the protein levels of COL1A1 among PG alone and PG with nicotine exposure in WT and nAChR α7 KO mice. PG with or without nicotine exposure reduced the protein abundance of fibronectin in WT female and nAChR α7 KO mice compared to air control (Figure 6A). However, decreased fibronectin protein level was observed in the PG-exposed WT male mice, and there was no alteration in nAChR α7 KO male mice among all the different exposure conditions (Figure 6B).

### Sub-chronic e-cig exposure in lung epithelial cell-specific nAChR α7 deletion protects against inflammation

Considering the inflammatory responses seen in WT and nAChR α7 KO mice, we exposed nAChR α7 lung epithelial cell-specific KO mice to e-cig with or without nicotine to determine the role of nAChR α7 in the lung epithelium. Surprisingly, we did not see any significant changes in pro-inflammatory cytokines following PG with nicotine exposure in nAChR α7 epithelial cell-specific KO mice. However, IL-5, MCP-1, KC, Eotaxin, GM-CSF, and G-CSF levels were significantly increased in PG alone group when compared to air control, and these cytokines were inhibited in nAChR α7 epithelial cell-specific KO mice (Additional File 1: Figure S4A). The rest of the cytokines from BALF showed no changes among all the different exposure groups in nAChR α7 epithelial cell-specific KO mice (Additional File 1: Figure S4B–C).

## Discussion

The popularity of e-cig vaping has been rising recently in the United States, and hence the health concerns about vaping have recently attracted public attention [27]. Our previous studies have shown that acute exposure to e-cigarettes could cause inflammatory responses, oxidative stress, and ECM remodeling [8, 11, 28]. Although in past, we have shown the health risks caused by acute e-cig exposures in mice [8, 11], sub-chronic or chronic effects of e-cig exposure-induced toxicity and respiratory health effects remain elusive. Based on our knowledge, no study has yet shown that long-term exposure of e-cig aerosol can result in persistent dysregulated repair/ECM remodeling and inflammatory responses implicating the role of nAChR α7 receptor in the lung.

Similar to our acute exposure results, e-cig aerosol with nicotine induced inflammatory cell counts in BALF compared to air control, especially increase in macrophages and CD4a^+^/CD8a^+^ T-lymphocytes. Previous report has demonstrated that two weeks of e-cig (with nicotine) exposure increased the number of macrophages but not neutrophils, which supports our findings [29]. Another study reported that acute exposure of e-cig aerosol increased total cell and macrophage counts; even the vehicle exposure alone showed a trend towards increase in total cells and macrophages [30]. Based on our results, e-cig exposure with or without nicotine causes increased inflammatory cellular influx into the lungs (BALF). PG alone (vehicle) exposure was considered safe by e-cig users, but our data reveals that sub-chronic e-cig exposure with PG (without nicotine) induced lung inflammation. The genetic ablation of nAChR α7 protects against e-cig exposure induced increased in macrophages and CD4a^+^/CD8a^+^ T-lymphocytes. Probably, e-cig exposure containing PG with nicotine causes lung inflammation/remodeling response via the nAChR α7 receptor mediated signaling pathway, and blocking the receptor could attenuate nicotine-dependent effects in the lungs. Prior reports show that activation of nAChR can also inhibit inflammation [31], and we have observed increased neutrophils and CD4a^+^/CD8a^+^ T-lymphocytes when exposed to PG alone in nAChR α7 KO mice. The nAChR deletion may promote inflammation independent of nicotine exposure in the lungs. The chemical derived from e-cig aerosols containing PG, such as formaldehyde, acetaldehyde, and methylglyoxal, in nicotine-free aerosol [32], may result in increased inflammation. As reported previously, both e-cig with or without nicotine caused infiltration of inflammatory cells and pro-inflammatory mediators that are key players for causing lung inflammatory response [8, 11, 30]. As an agonist of nAChR α7, nicotine might be pose both pro-inflammatory and anti-inflammatory roles, and further research is needed to study the relationship between nicotine and nicotinic receptors in the context of sub-chronic e-cig exposure.

Apart from the inflammatory cells, some pro-inflammatory mediators in BALF were significantly increased following e-cig exposure with nicotine. Specific macrophage-mediated pro-inflammatory cytokines such as IL-1α, MCP-1, and GM-CSF, were increased in BALF after PG with nicotine exposure in a nAChR α7-dependent manner. However, a few other macrophage-driven cytokines, IL-1β, TNF-α, and MIP-1β, does not show nAChR α7 dependency. Our results paralleled reports that e-cig aerosol containing nicotine induced cytotoxicity in alveolar macrophage and significant release of inflammatory mediators (TNF-α, CXCL-8, MCP-1, and IL-6) [33]. Additional studies have shown specific T-lymphocytes-related cytokines/chemokines (IL-2, IL-9, IFN-γ, and RANTES) and their role in inflammatory signaling [34–37], and our results indicated that these altered cytokines response were nAChR-dependent in e-cig exposed mouse lungs. Mice exposed to PG with nicotine show increased IL-5 release in a nAChR independent manner. Another report shows increased IL-4, IL-5, and IL-13 levels following e-cig containing nicotine when challenged to ovalbumin treated mice resulting in exacerbation of allergic airway inflammation [38]. The increased IFN-γ production has been related to cytotoxicity and infiltration of CD8a^+^ T-cells [39, 40]. These altered pro-inflammatory cytokines response and cellular influx in the lung are interconnected to each other partly due to nAChR α7 mediated signaling. It is possible that nAChR α7 deletion could attenuate the inflammatory responses induced by e-cig aerosol contained nicotine in the lungs. However, PG-induced increase in CD4a^+^/CD8a^+^ T-lymphocytes does not corroborate with the cytokine levels. Future studies will address immune cell-type specific role in pulmonary toxicity of e-cig with and without nicotine during long-term exposure in mice.

We measured the mRNA expression using the mouse myeloid innate immunity panel from Nanostring technology to screen the potentially affected target genes. Our results showed altered expression of SKIL and LDLR in PG with nicotine-exposed WT mice, but the same genes remain unaffected in nAChR α7 KO exposed groups. These target genes have been shown to play vital role in TGFβ/SMAD canonical signaling and TGFβ-induced epithelial–mesenchymal transition (EMT) may be inhibited by SKIL binding to SMAD [41, 42]In this study, we found increased protein abundance of PAI-1 in WT mice exposed to PG with nicotine, but PAI-1 was further reduced in nAChR α7 KO mice. Our results suggest that e-cig exposure with PG nicotine may affect SKIL-TGFβ-PAI-1 axis in the lung possibly through nAChR α7 activation. The other important target significantly altered by PG with nicotine is LDLR gene. Overexpression of LDLR is capable of promoting macrophage differentiation and exaggerated inflammatory response and silencing LDLR results in reduced inflammatory responses in THP-1 cells [43]. In our study, LDLR was reduced in PG with nicotine exposed WT mice, while LDLR transcript levels remain unchanged in both PG with and without nicotine exposed nAChR α7 KO groups. These findings suggest that nAChR α7 may be an important gateway that affects specific inflammatory response/ECM remodeling target genes during sub-chronic e-cig exposure with nicotine.

To further study the inflammatory responses, we measured the protein abundance of NF-κB (p50/p105) in both female and male mice. The activation of NF-κB is well documented in different lung injury models [44] including cigarette smoke-induced inflammation, wheras research on e-cig aerosol induced NF-κB activation remain less explored [45–48]. Although, the anti-inflammatory effects (i.e. inhibition of NF-κB signaling) via activation of nAChRs is well-known and has been studied previously, nicotine has been shown induce inflammation through activation of the NF-κB signaling pathway [31, 49]. Our findings suggest that e-cig aerosol containing nicotine increased p50 and p105 protein abundance in female mice in a nAChR α7-dependent manner. We believe nicotine is a potent inducer of altered lung inflammation via activated nAChR α7 compared to the anti-inflammatory effects regulated by nAChR-related signaling. We found lack of nAChR α7 showing attenuation of inflammatory response following e-cig exposure with nicotine. However, nicotine-free e-cig exposure induced p105 protein levels, which suggest this subunit may act through an alternative pathway that may be independent of nAChR α7 signaling. We for the first time show that lung inflammation caused by e-cig aerosol containing nicotine is nAChR α7-dependent, preferentially in a sex-specific manner. However, more detailed studies are needed to further illustrate the exact mechanism by which sex-differences in lung inflammatory responses occurs following e-cig exposure through cell-type specific nAChR α7-mediated signaling.

As we recently reported, acute exposure of e-cig aerosol with or without nicotine resulted in dysregulated repair and ECM remodeling [8]. We noticed similar effects in our sub-chronic exposure model as well. E-cig aerosol with or without nicotine alters MMPs and ECM remodeling proteins in a sex-dependent manner, which are nAChR α7 receptor independent. It is known that nicotine exposure causes harmful respiratory effects, such as airway remodeling and airspace enlargement [50, 51], which is a key feature of emphysema and COPD [52–54]. However, dysregulation of MMPs serves not only as a factor for ECM remodeling, but also as a marker of inflammation mediated by dysregulated macrophages. Based on our results, nicotine-free e-cig aerosol induced augmented MMPs dysregulation than e-cig aerosol with nicotine. Increased MMP2/MMP12, along with decreased MMP9/MMP8, might suggest a compensatory feedback loop [55]. We observed that ACE2 is induced by e-cig with nicotine exposure, whereas the levels is decreased in nAChR α7-deficient micesuggesting that ACE2 upregulation by nicotine is mediated by nAChR α7. It is thought that ACE2 is upregulated by smoking, as well as its expression level is increased in smokers and COPD patients [56, 57], as well as served as the entry gate for the novel coronavirus (SARS-CoV-2) [58]. Hence, e-cig with nicotine inhalation would promote SARS-CoV-2 infection, and nAChR α7 deletion and downregulation of ACE2 may have some ramifications on the virus infection.

One of the major substrates of MMPs is ECM related proteins. From our previous study, mice exposed to e-cig aerosols for 3 days showed dysregulated repair/ECM remodeling both at the mRNA and protein level in a sex-dependent manner [8]. The current study shows that sub-chronic e-cig exposure with or without nicotine affects ECM remodeling to a certain extent dependent on nAChR α7 with some sex-specific phenotypes. We have observed increased PAI-1 only in WT male mice exposed to PG with nicotine in a nAChR α7-dependent manner, and PAI-1 is a primary ECM regulator and pro-fibrotic marker [59]. Therefore, increased PAI-1 following inhalation of e-cig aerosol with nicotine indicates that e-cig vapor containing nicotine could increase risk of chronic fibrotic disease, implicating crucial role of nAChR α7-related signaling. Some other ECM proteins, such as type 1 collagen and fibronectin were altered after exposure of e-cig aerosol with or without nicotine. ECM proteins affected by e-cig exposure are associated with dysregulated wound healing and fibrotic disease [60]. The observed increase in COL1A2 following e-cig with PG alone exposure in both male and female mice demonstrates the significant health risk imposed by PG alone, in addition to the risks associated with PG nicotine. As mentioned above, nicotine might not be the only target that can activate nAChR α7, and PG alone via other indirectly mechanisms could activate nicotinic receptors, which may be in agreement with our previous study [8, 19]. It is well known that collagen and fibronectin are required when the wound healing process happens and delayed wound healing were observed when epithelial cells were unable to synthesize fibronectin [61]. We found down-regulation of collagen and fibronectin following e-cig exposure, which will make lungs more vulnerable later in life when exposed to other noxious gases and environmental toxicants. Further studies are required to understand the mechanisms underlying how e-cig exposures may induce damaging responses, leading to lung disease and injury.

Based on our findings, long-term studies are needed to provide further insights on how nAChRα7 dependency plays an important cell-type specific role in the lung (epithelial and fibroblast) during e-cig exposure, and its involvement in ACE2 regulation. We know that several factors, such as duration of exposure, route of exposure, dose, e-liquid composition, setting used for delivering the e-cig aerosols, concentration of PG mixture and nicotine, including mouse strain, background, sex of mice may equally contribute to the difference and phenotype observed in the measure outcomes. Chronic e-cig exposure are underway that will help us to understand how these signaling mechanisms altered by e-cig exposure with and without nicotine may contribute to the respiratory toxicity observed in mice could translate to clinical relevance in human e-cig users. Ongoing human studies are being carried out to identify potential targets and toxicity biomarkers that may relates to human respiratory health effects of e-cigarettes.

## Conclusion

In conclusion, nAChR α7 ablation attenuates the inflammation induced by sub-chronic e-cig exposure may be partly due to e-cig induced ECM remodeling/dysregulated repair response in the lung. In a sub-chronic e-cig exposure, nAChR α7 plays a novel role in an anti-inflammatory response induced by nicotine. We show a significant difference in terms of altered genes that belong to inflammation, dysregulated repair and ECM remodeling in WT mice compared to nAChR α7 KO mice when exposed to PG with nicotine. The differential inflammatory cell counts and pro-inflammatory cytokines in BALF were comparable to our previous findings. Hence, nAChR α7 may play a vital role in the inflammatory responses induced by nicotine-containing e-cig aerosols. Our data suggest that ECM remodeling/dysregulated repair caused by e-cig exposure is mediated by nAChR α7, and occurs in a sex-dependent manner. Overall, e-cig aerosol with nicotine causes lung inflammation due to altered dysregulated repair response and ECM remodeling mediated by nAChR α7. Deletion of nAChR α7 may possibly protect against lung inflammation and injury by attenuating associated signaling targets for ECM remodeling. Hence, nAChR α7 is considered a valid target for inflammation induced by e-cig aerosol with nicotine in the lung.

## Supplementary Material

Supplement

Supplement

## Abbreviations

E-cig: Electronic cigarette
ECM: extracellular matrix
nAChRs: Nicotinic Acetylcholine Receptors
WT: wild-type
nAChRα7 KO: nAChRα7 knockout
nAChRα7 CreCC10: lung epithelial-cell-specific nAChRα7 KO
PG: propylene glycol
VG: vegetable glycerin
BALF: Bronchoalveolar lavage fluids
MMPs: matrix metalloproteinases
IPF: idiopathic pulmonary fibrosis
COPD: chronic obstructive pulmonary disease
CNS: central nervous system
LPSs: lipopolysaccharides
Glu: glucose
Hb: hemoglobin
Hct: hematocrit
AO/PI: acridine orange propidium iodide
CCL9: Chemokine ligand 9
KLF4: Kruppel-like factor 4
DUSP1: Dual Specificity Phosphatase 1
BTLA: B and T Lymphocyte Associated
SMAD7: SMAD Family Member 7
NF-κB: nuclear factor kappa-light-chain-enhancer of activated B cells
PAI-1: Plasminogen activator inhibitor-1
COL1A1: type 1 collagens chain 1
COL1A2: type 1 collagens chain 2
ACE2: Angiotensin-converting enzyme 2
SARS-CoV2: Severe acute respiratory syndrome coronavirus 2
