# Supplementary material for: E-cigarette-Induced Pulmonary Inflammation and Dysregulated Repair are Mediated by nAChR α7 Receptor: Role of nAChR α7 in ACE2 Covid-19 receptor regulation
Source: Res Sq. 2020 May 18:rs.2.23829. Preprint. [Version 2] doi: 10.21203/rs.2.23829/v2 (PMC7336696; doi:10.21203/rs.2.23829/v2)
Supplement: Supplement [file Additionalfile2suppinfoTable.docx]

**Table S1. Sub-chronic e-cig exposure using PG with or without nicotine altered blood gas but not hematology.**

| **Genotype** | **Exposure**  **condition** | **pH** | **pCO_2_ (mmHg)** | **pO_2_ (mmHg)** | **HCO_3_ (mmol/L)** | **TCO_2_ (mmol/L)** | **sO2 (%)** | **Glu (mg/dl)** | **Hct (%PCV)** | **Hb (g/dl)** |
| --- | --- | --- | --- | --- | --- | --- | --- | --- | --- | --- |
| **C57BL/6J (WT)** | **PG** | 7.34±0.03 | 43.52±3.73 | 36.87±2.35 | 23.97±2.91 | 25.12±2.29 | 67.12±4.18 | 192.62±20.66 | 44.62±0.92 | 15.17±0.30 |
|  | **PG+Nic** | 7.28±0.07^#^ | 42.3±4.17 | 44.0±9.03*^#^ | 20.0±3.64^#^ | 21.37±3.24*^#^ | 71.75±9.26 | 194.25±40.69 | 45.37±1.84 | 15.42± 0.61 |
| **nAChR7 KO** | **PG** | 7.32±0.05 | 44.42±4.27 | 38.87±7.72 | 22.82±3.04 | 24.12±2.94 | 67.0±8.97 | 176.13±23.91 | 43.37±1.84 | 14.75±0.62 |
|  | **PG+Nic** | 7.32±0.08 | 43.45±2.35 | 36.0±4.03 | 23.07±3.11 | 24.37±3.20 | 63.62±9.21 | 166.87±50.10 | 44.87±1.64 | 15.26±0.53 |

Blood gas analysis and hematology test results using venous blood samples by iSTAT system in nAChR7 KO & C57BL/6J mice. Data are presented as mean± SEM (n=3, * P < 0.05, significant compared between nAChR α7 KO vs WT in PG+Nic group, # P < 0.05, significant compared between WT-PG group vs WT-PG+Nic.)

**Table S2. Sub-chronic e-cig exposure using PG with nicotine increased pro-inflammatory cytokines in BALF.**

| Cytokines | WT | | | nAChR α7 KO | | |
| --- | --- | --- | --- | --- | --- | --- |
|  | Air | PG | PG+Nic | Air | PG | PG+Nic |
| IL-1β | 1.5 ± 0.1 | 1.5 ± 0.17 | 2 ± 0.065 * | 1.4 ± 0.077 | 1.4 ± 0.078 | 1.9 ± 0.021 * |
| IL-3 | 0.21 ± 0.031 | 0.32 ± 0.068 | 0.33 ± 0.029 | 0.2 ± 0.058 | 0.24 ± 0.028 | 0.21 ± 0.02 |
| IL-4 | 0.55 ± 0.067 | 0.68 ± 0.042 | 0.8 ± 0.091 | 0.59 ± 0.062 | 0.64 ± 0.042 | 0.7 ± 0.09 |
| IL-6 | 1.1 ± 0.07 | 1.2 ± 0.12 | 1.6 ± 0.055 * | 1.1 ± 0.18 | 1 ± 0.066 | 1.3 ± 0.13 |
| IL-10 | 9.5 ± 0.49 | 11 ± 1.1 | 12 ± 0.44 | 9.9 ± 0.58 | 11 ± 0.47 | 11 ± 0.71 |
| IL-12p40 | 52 ± 4.8 | 61 ± 7.6 | 47 ± 5.3 | 70 ± 11 | 93 ± 24 | 44 ± 2.5 |
| IL-12p70 | 18 ± 1.3 | 22 ± 6.3 | 31 ± 2 ** | 19 ± 3.2 | 17 ± 1.3 | 23 ± 3.6 |
| IL-13 | 55 ± 4.4 | 57 ± 4.9 | 68 ± 1.8 ** | 49 ± 4.3 | 52 ± 2.8 | 57 ± 5.5 |
| IL-17A | 2.5 ± 0.15 | 3.1 ± 0.31 | 3.3 ± 0.095 | 3.5 ± 0.43 | 3 ± 0.13 | 3 ± 0.23 |
| KC | 19 ± 0.14 | 20 ± 1.1 | 24 ± 0.71 ** | 23 ± 3 | 19 ± 1.4 | 21 ± 1.5 |
| G-CSF | 4.7 ± 0.26 | 5.9 ± 0.54 | 5.4 ± 0.25 | 4.8 ± 0.34 | 5.5 ± 0.32 | 5 ± 0.3 |
| Eotaxin | 50 ± 3 | 62 ± 5.9 | 69 ± 1.9 * | 51 ± 2 | 61 ± 3.5 | 53 ± 4.1 **^#^** |
| MIP-1α | 0.72 ± 0.1 | 0.79 ± 0.091 | 0.99 ± 0.1 | 1.6 ± 0.42 | 0.84 ± 0.12 | 1 ± 0.074 |

Bio-Plex Pro mouse cytokine 23-plex assay kit (Bio-Rad) was used to determine levels of pro-inflammatory cytokines/chemokines in BALF from mice exposed to e-cig for 30 days (2 hrs/day). Levels of IL-1β, IL-3, IL-4, IL-6, IL-10, IL-12p40, IL-12p70, IL-13, IL-17A, KC, G-CSF, Eotaxin, and MIP1-α are listed. Data are shown as mean ± SEM (n=6-10/group; equal number of male and female mice). (* P < 0.05, ** P < 0.01 compared with air control in mice of same genotype, # P < 0.05 compared with PG+Nic exposed WT mice.)
